# Agreement of Parathyroid Hormone Status Measured by Intact and Biointact Parathyroid Hormone Assays among Chronic Kidney Disease Patients and Its Correlation with Bone Turnover Parameters

**DOI:** 10.21315/mjms2023.30.2.6

**Published:** 2023-04-18

**Authors:** Saidah Madihah Hashim, Tuan Salwani Tuan Ismail, Noor Azlin Azraini Che Soh, Mahaya Che Mat, Zuad Firdaus Rapiah, Noorazliyana Shafii, Nur Karyatee Kassim, Najib Majdi Yaacob

**Affiliations:** 1 Chemical Pathology Unit, Hospital Raja Perempuan Zainab II, Kelantan, Malaysia; 2 Department of Chemical Pathology, School of Medical Sciences, Universiti Sains Malaysia, Kelantan, Malaysia; 3 Nephrology Unit, Department of Medical, Hospital Raja Perempuan Zainab II, Kelantan, Malaysia; 4 School of Dental Sciences, Universiti Sains Malaysia, Kelantan, Malaysia; 5 Biostatistics and Research Methodology Unit, Universiti Sains Malaysia, Kelantan, Malaysia

**Keywords:** PTH assays, intact PTH, biointact PTH, bone turnover markers, chronic kidney disease, metabolic bone diseases

## Abstract

**Background:**

This study aimed to determine the agreement between intact parathyroid hormone (iPTH) and biointact parathyroid hormone (bio-PTH) assays and to correlate them with bone markers.

**Methods:**

This cross-sectional study included 180 patients with chronic kidney disease (CKD) stages 3b, 4 and 5D. We measured their iPTH, bio-PTH, 25-hydroxyvitaminD (25(OH)D), C-terminal telopeptide collagen (CTX), procollagen 1 intact N-terminal propeptide (P1NP), calcium, phosphate and alkaline phosphatase (ALP).

**Results:**

Higher iPTH than bio-PTH concentrations were seen in CKD stages 3b, 4 and 5D (58[62] versus 55[67] pg/mL, 94[85] versus 85[76] pg/mL and 378[481] versus 252[280] pg/mL, respectively). Both PTH assays showed good agreement among all the subjects, with an intraclass correlation coefficient of 0.832 (*P*-value < 0.001). The Passing-Bablok showed that the equation for the bio-PTH was PTH = 0.64 iPTH + 15.80, with *r* = 0.99. The Bland-Altman plots showed increased bias with an increasing PTH concentration. Both PTH assays showed a high positive correlation with CTX and P1NP, a moderate correlation with phosphate, a low correlation with ALP and calcium, and a negligible correlation with phosphate and 25(OH)D.

**Conclusion:**

The iPTH and bio-PTH assays were in agreement, but their bias increased with the PTH concentration. The unacceptable large bias indicates that the two assays cannot be used interchangeably. They had a variable correlation with the bone parameters.

## Introduction

Chronic kidney disease (CKD) has been growing in prevalence in recent years. Despite available guidelines for reducing CKD complications, cases of CKD with bone mineral disorder are still common. CKD-mineral bone disease (CKD-MBD) is defined by the presence of any of the following conditions: biochemical abnormalities, abnormalities in bone turnover and mineralisation, and/or soft tissue and vascular calcification (1). The bone disease component of CKD-MBD may cause fractures and bone pain (2). For biochemical abnormalities, the Kidney Disease Improving Global Outcomes (KDIGO) guidelines 2017 recommend regular monitoring of serum calcium, phosphate, parathyroid hormone (PTH) and alkaline phosphatase (ALP) starting from CKD stage 3. CKD cases with estimated glomerular filtration rates (eGFRs) of 44–59 mL/min/1.73 m^2^, 15–29 mL/min/1.73 m^2^ and less than 15 mL/min/1.73 m^2^ are classified as stages 3b, 4 and 5, respectively. If the eGFR is less than 60 mL/min/1.73 m^2^, the fracture prevalence rate more than doubles; and if the kidney function keeps declining, the fracture risk further increases (3–5). Evidence shows that CKD-MBD patients are also predisposed to cardiovascular calcification, with associated high morbidity and mortality rates (2, 6). Therefore, more efforts are needed for earlier CKD-MBD detection and prevention.

PTH is elevated in CKD due to hypocalcaemia caused by impaired vitamin D activity and phosphate retention when the renal function is reduced. Furthermore, PTH is an important regulator of the calcium and phosphate levels in the human body and should be maintained within the target range. From the clinical viewpoint, PTH measurement is crucial for CKD-MBD diagnosis and monitoring, and is involved in therapeutic decision making. Available PTH assays are second-generation intact PTH (iPTH) assay and third-generation PTH assay (7), also known as biointact PTH (bio-PTH), whole PTH (wPTH) or biologically active PTH assay.

iPTH assay has an established reference range and a high volume of clinical data on its usefulness, besides being quoted in the KDIGO guidelines (1). However, it was found to also detect 7–84 PTH fragments, which decrease serum calcium and urine phosphate and inhibit bone resorption, unlike the 1–84 bio-PTH fragments (8). This N-truncated terminal fragment was found to be renally excreted and accumulated as the renal function deteriorates, and to lead to overestimation of the PTH measurement in patients (9, 10). Bio-PTH assay detects the full-length 1–84 bio-PTH fragments, but an internationally accepted reference range has not yet been established and clinical data on its usefulness is lacking compared to iPTH assay (8).

Studies have proven the superiority of bio-PTH assay to iPTH assay. Lehmann et al. (11) compared the PTH concentrations measured with iPTH and bio-PTH assays and found that both assays discriminate excellently between patients with high or low CKD-MBD turnover. However, bio-PTH assay discriminated slightly better between high and low bone turnover in CKD-MBD patients. Melamed et al. (12) found a higher bio-PTH concentration significantly associated with mortality in dialysis patients, unlike the iPTH concentration. O’Flaherty et al. (13), Tan et al. (14), Inaba et al. (15), Einbinder et al. (16) and Dupuy et al. (17) agreed that bio-PTH assays correlated well with iPTH assays, but the PTH concentration in the former was lower than that in the latter. However, those studies were conducted in a specific CKD stage. There are limited data correlating iPTH and bio-PTH in all CKD stages and in pre-dialysis and dialysis patients.

Bone biopsy is the gold standard for bone turnover evaluation. However, its high cost, invasiveness and lack of local expertise, besides the burden of repeat biopsy for each bone turnover monitoring, limit its usefulness. KDIGO 2017 recommends bone biopsy only if the result will change the treatment decision. Bone resorption and bone formation must be tightly coupled to maintain the bone mass; otherwise, they will cause MBD. Bone formation markers include N-terminal propeptide of type I procollagen (PINP) and bone resorption markers include C-terminal telopeptide of type I collagen (CTX). Bone turnover markers were found to be more practical, cost-effective and reliable in determining the bone turnover rate. This study evaluated the agreement between iPTH and bio-PTH assays in CKD stages 3, 4 and 5D (dialysis) and determined the correlation between iPTH and bio-PTH assays and, PINP and CTX.

## Methods

### Study Participants

This cross-sectional study was conducted at the Nephrology Clinic of the Haemodialysis and Continuous Ambulatory Peritoneal Dialysis (CAPD) Unit of Hospital Raja Perempuan Zainab II (HRPZ II) in Kota Bharu, Kelantan, Malaysia, a state hospital on the east coast of Malaysia, with a population of 1.9 million in 2020 (18). The study participants were recruited from 1 January 2020 to 31 December 2020. This study was based on the guidelines for good clinical practice.

The sample size was calculated using the intraclass correlation coefficient (ICC) formula, with a type I error of 0.5 (two tails), a power of 0.8, two measurements per subject, a smallest acceptable ICC of 0.6 and an expected ICC of 0.98, a confidence level of 0.95, and a provision of estimate of 0.05. The required sample size was 48. Anticipating 20% dropouts, the corrected sample size was 60. For each of the CKD stages 3, 4 and 5D, the sample size was 60, so the total sample size was 180. Stages 3 and above were selected because the measurement of PTH and other bone parameters is monitored from stage 3. Stage 5D was chosen because we wanted to compare the performance of the assays on dialysis patients. Using simple random sampling, we obtained the names of the patients scheduled for routine blood taking from the clinic or the dialysis unit. Cases were defined as patients aged above 18 years with CKD stages 3, 4 or 5D based on the Chronic Kidney Disease-Epidemiology Collaboration (CKD-EPI) formula to estimate the GFR (19). Individuals with acute kidney injury and those with a history of parathyroidectomy were excluded. The patients were screened based on the inclusion and exclusion criteria using a hospital computer software programme and direct inquiry during the blood-taking session. On the day of the blood taking and dialysis, every third name on the list was selected to be a subject of this study.

### Biochemical Measurements

Six millilitre of pre-dialysed fasting venous blood samples was collected in a gel separator tube. The blood was centrifuged at 3,500 rpm for 8 min at 25 °C. Then, the samples were aliquoted and stored at −80 °C for batch analysis. The serum calcium, phosphate and ALP levels were measured in the serums via spectrophotometric analysis using Beckman Coulter AU 5810. The measured serum 25-hydroxyvitamin D (25(OH)D), P1NP and CTX were tested with immunoassay techniques using Cobas e601 (Roche Diagnostics GmbH, Germany), whereas the iPTH and bio-PTH (1–84) concentrations were measured using the Cobas Elecsys PTH and PTH (1–84) immunoassays, which employ the sandwich test principle electrochemiluminescence immunoassay (ECLIA).

The Cobas Elecsys PTH assay used a biotinylated monoclonal antibody to react to the N-terminal fragment (1–37 amino acids) and a monoclonal antibody labeled with a ruthenium complex to react to the C-terminal fragment (38–84 amino acids). The Cobas Elecsys PTH (1–84) assay used a biotinylated monoclonal antibody to react to the N-terminal fragment PTH (1–5 amino acids) and a monoclonal antibody labeled with a ruthenium complex to react to the C-terminal fragment PTH (54–59). Hence, it specifically measured the bioactive molecule of PTH and PTH (1–84). iPTH assay has a coefficient of variation (CV) for precision and repeatability of 2.5%–3.4% and 1.1%–2.0%, respectively, whereas bio-PTH assay has a CV of 2.4%–3.0% and a repeatability of 0.7%–3.5%.

### Statistical Analysis

Data were expressed as mean (standard deviation [SD]) values when the distribution was normal and as median with interquartile range (IQR) values otherwise. Three analyses were conducted to compare the PTH assays. First, the two-way random effects, absolute agreement and single-rater measurement of ICCs were calculated focused on the agreement and correlation of the PTH assays (19). ICC values of < 0.5 were considered as poor; 0.5–0.75, moderate; 0.75–0.9, good; and > 0.90 of excellent reliability (20). Bland-Altman analysis was performed to assess bias across the measurement range. Systematic bias between the PTH assays was determined using Passing-Bablok regression analysis. Associations between PTH (second- and third-generation assays) and bone turnover markers (CTX and P1NP), 25(OH) D, calcium, phosphate and ALP were evaluated using Spearman rho correlation analysis. The *r*-value indicated the strength of the monotonic relationship between the analytes tested. A value closer to +1 indicated a stronger correlation; −1, a stronger negative correlation; and 0, no correlation. A value of 0.90–1.00 showed a very high correlation; 0.70–0.80, high correlation; 0.50–0.70, moderate correlation; 0.30–0.50, low correlation; and 0.00–0.30, negligible correlation (21).

Statistical analyses and calculations were carried out with IBM SPSS version 24.0 for normality testing, descriptive statistics, ICC measurement and Spearman rho regression analysis. Passing-Bablok regression and Bland-Altman analysis were performed using the method comparison regression package in the *R* software version 4.0.3.

## Results

### General Characteristic of the Study Subjects

A total of 180 patients enrolled in this study. They were aged 22 years old–84 years old with a median of 58 and an IQR of 18; 51.7% were men and 48.3%, women; and 96.6% were Malay and 3.4%, Chinese and Siamese. Their baseline laboratory characteristics are shown in Table 1. The iPTH levels were higher than the bio-PTH levels in each stage and the PTH concentration increased with the CKD progression.

### Agreement and Correlation between the Intact Parathyroid Hormone and Biointact Parathyroid Hormone Assays

**Figure 1:**
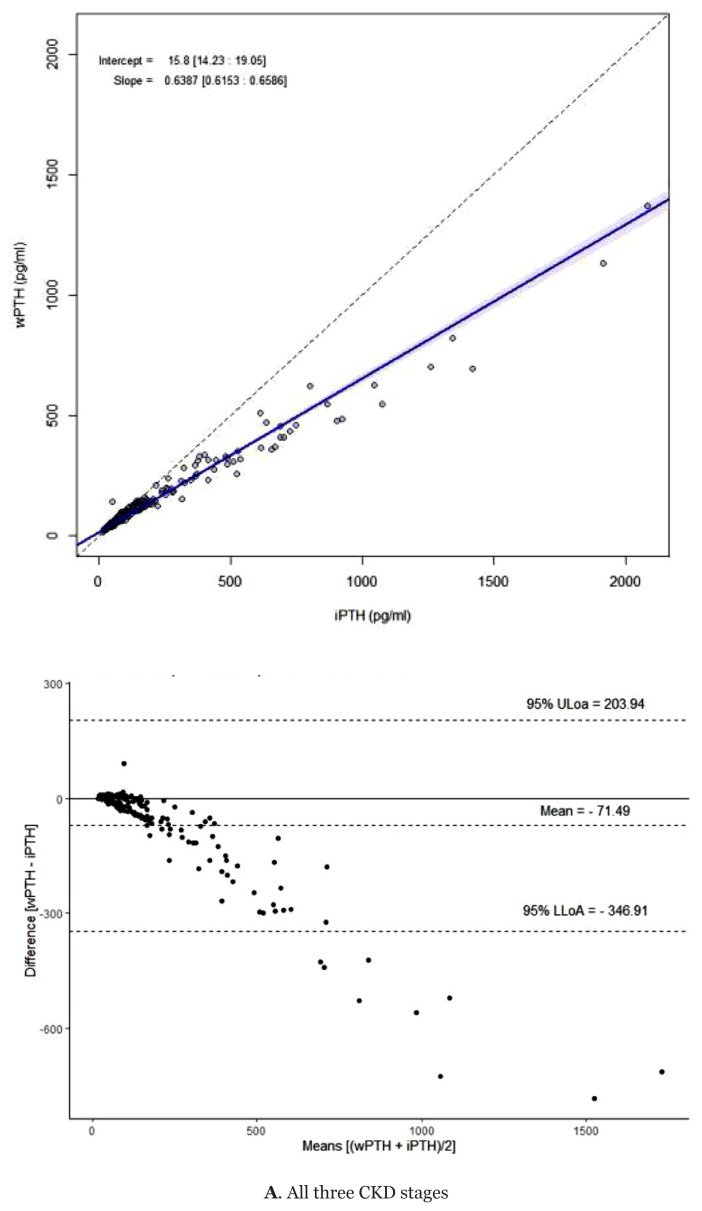

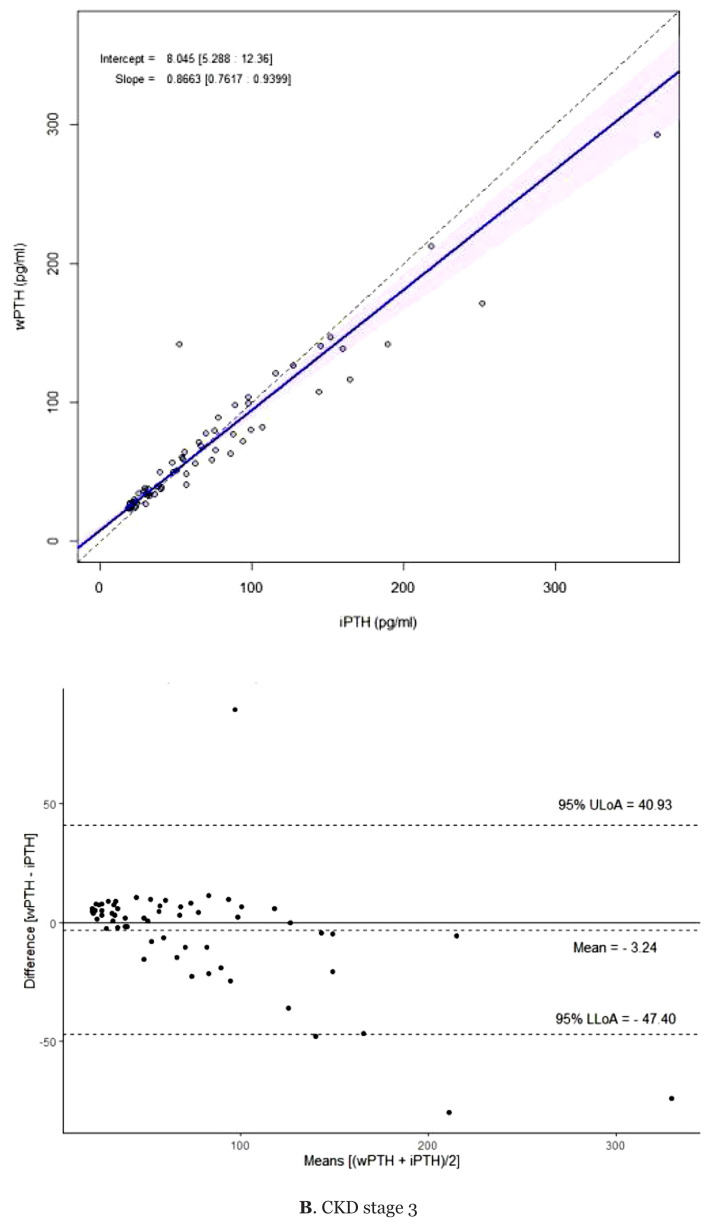

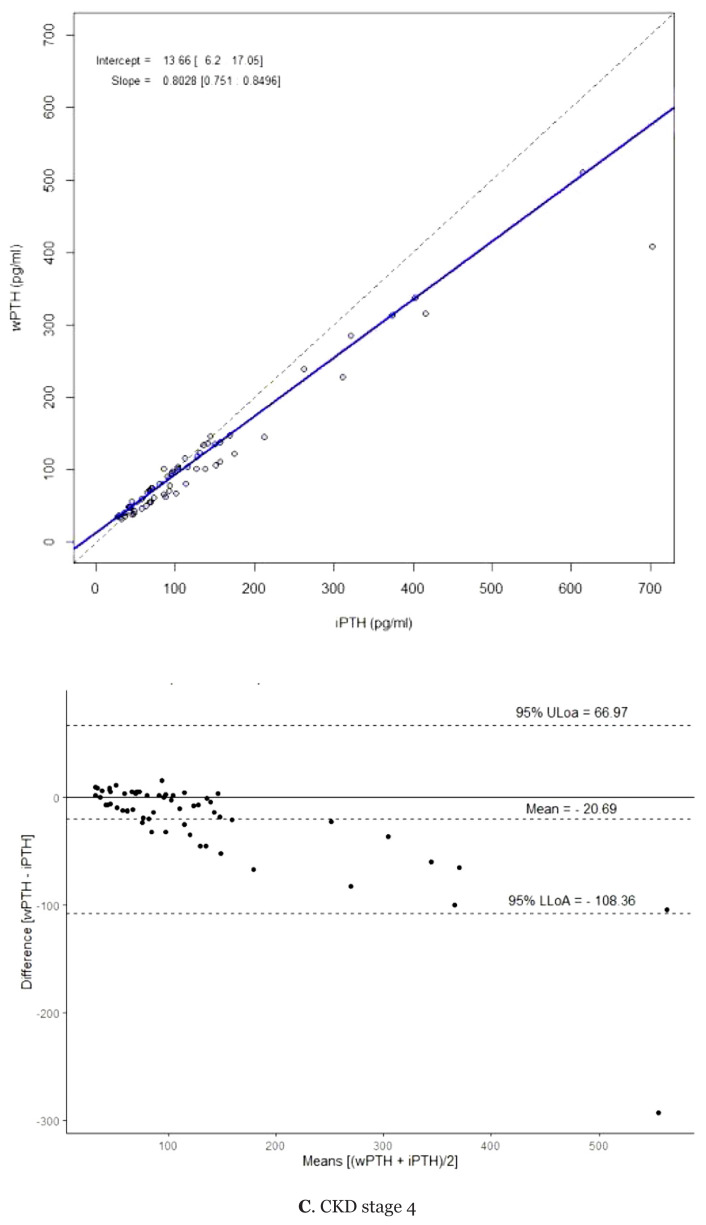

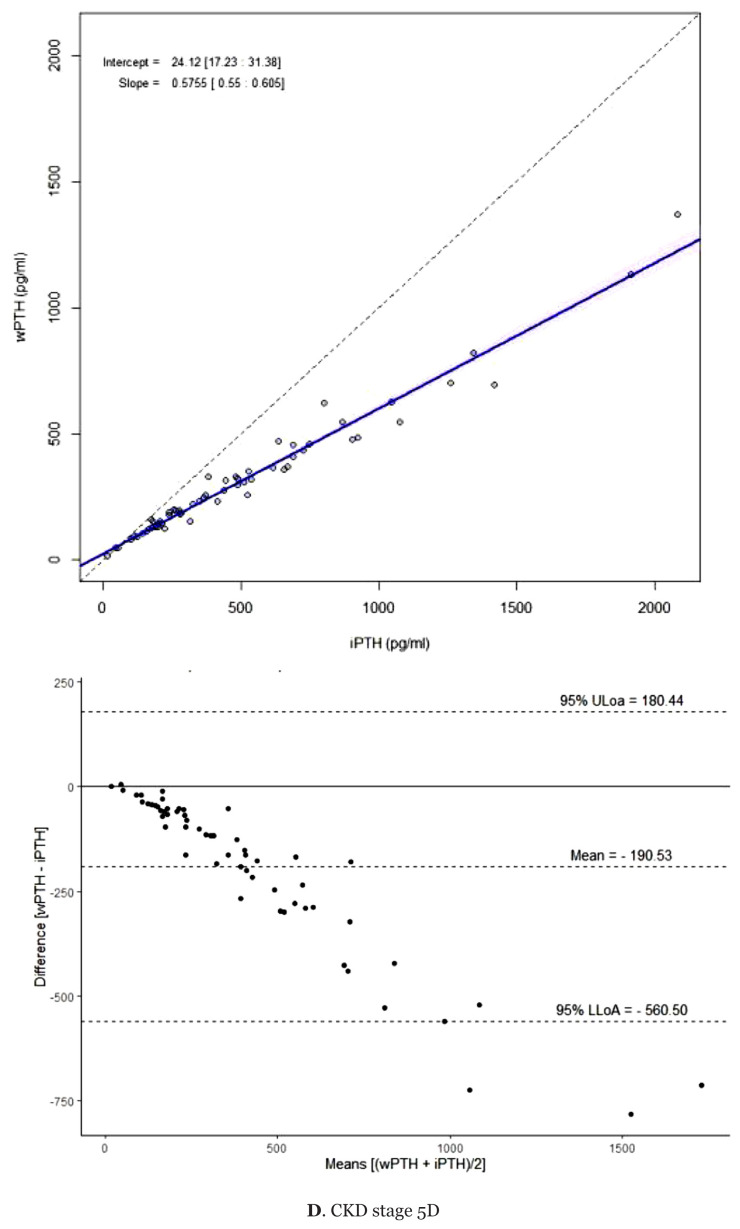
Comparison of PTH concentrations (pg/L) of iPTH and biointact PTH using Passing-Bablok regression scatter plot and Bland-Altman percentage difference plot assays according to: **A**. all CKD stages, **B**. CKD stage 3, **C**. CKD stage 4, **D**. CKD stage 5D

There was generally good agreement between the two PTH assays (Table 2). The ICC values for CKD stages 3b–5D, CKD stage 3b, CKD stage 4 and CKD stage 5D were 0.832, 0.926, 0.912 and 0.749, respectively. These show that the degree of reliability decreased as the CKD stage increased, from excellent reliability in stages 3b and 4 to moderate reliability in stage 5D. The PTH assay methods were also compared using Passing-Bablok regression and Bland-Altman analysis (Figure 1). The figure shows that the result discrepancies increased with the PTH concentration. The PTH concentrations in the dialysed patients determined by bio-PTH assays were approximately 30% lower than those measured by iPTH assays. The results of the analysis of the Passing-Bablok regression parameters (slope and intercept) and of the Bland-Altman analysis with the mean difference and the limit of agreement (LOA) are shown in Table 3.

### Biases of the Intact Parathyroid Hormone and Biointact Parathyroid Hormone Assays by Chronic Kidney Disease Stage

The correlation between the assays for the CKD stages were given by the Passing-Bablok regression equation bio-PTH = 0.64 iPTH + 15.80 and by the Bland-Altman analysis with an average bias of −71.49 pg/mL and a LOA of −346.91 to 203.94. Further stratification of the results according to the CKD severity showed that despite the good agreement between the assays, the bias increased with the PTH concentration and the CKD stage. The average bias in CKD stage 3b was −3.24 (LOA: −47.40 to 40.93) and in stages 4 and 5D, −20.69 (LOA: −108.36 to 66.97) and−190.53 (LOA: −560.50 to 179.44), respectively. Subsequent evaluation of both methods showed a bias beyond the clinical decision limit (Table 4).

### Correlation of the PTH Assays with the Bone Markers

Both PTH assays were significantly correlated with the bone markers and 25(OH) D, as shown by the *P*-value in Table 5. However, both PTH assays showed only a high positive significant correlation with CTX and P1NP, moderate with phosphate and low with ALP; a low negative correlation with calcium; and a negligible correlation with 25(OH)D.

## Discussion

This study compared the PTH level results obtained from the iPTH and bio-PTH assays among the patients with CKD stages 3 to 5D and determined the correlation of the PTH concentrations from both assays with the bone turnover markers. The demographic data showed that the median age was about the same across the CKD stages. As the CKD stage progressed, the PTH concentrations in both assays, phosphate, ALP, CTX and P1NP increased, and the calcium and 25(OH)D concentrations decreased. The PTH concentration measured by the bio-PTH assay was lower than that measured by the iPTH assay. This difference is explained not only by the iPTH assay’s detection of bio-PTH (1–84) fragments but also by its cross-reaction with other amino terminally truncated PTH fragments such as PTH (7–84), which is renally excreted and thus, accumulated with a decreasing eGFR.

It was also observed that when the patient had entered the dialysis stage, the circulating amino terminal truncated PTH fragments were as abundant as the bio-PTH (1–84) fragments. This led to a higher iPTH than bio-PTH measurement. We found that the PTH concentration obtained using iPTH assay was generally higher in all three CKD stages than that obtained using bio-PTH assay. There was good agreement and a positive correlation between the two assays, based on the ICC value and the Passing-Bablok regression. The bio-PTH concentration had a concurrent increment with the iPTH concentration increment, but the former was not as significant as the latter. The Passing-Bablok regression result agrees with that of O’Flaherty et al. (13) and of Einbinder et al. (16). To our knowledge, the agreement between the two assays has not been evaluated yet using the ICC.

The Bland-Altman plot showed a large bias with a wide LOA in CKD stage 5D. The bias between the two PTH assays was low at a lower PTH concentration and increased as the PTH concentration increased. Our findings conform with those of studies that compared iPTH and Roche bio-PTH assays. O’Flaherty et al. (13) and Dupuy et al. (17) showed that the bio-PTH levels were significantly lower than the iPTH levels, but they showed concordance among healthy populations and their bias increased with CKD progression. Einbinder et al. (16) evaluated the correlations and differences between iPTH and bio-PTH assays in CKD stages 3, 4 and 5, and found that the bio-PTH concentrations were consistently strongly correlated with, but significantly lower than, the iPTH concentrations. Similarly, Hecking et al. (22) compared two-generation Roche PTH assays among haemodialysis patients in Vienna and found that such assays had a strong positive correlation and that the bio-PTH level was roughly two-thirds lower than the iPTH level. In a study among CKD patients in different stages (23), the concentration of bio-PTH in dialysed patients was more than 40% lower than that of iPTH. This percentage is higher than in our study, in which the bio-PTH concentration was 30% lower than the iPTH concentration among dialysed patients.

The European Federation of Clinical Chemistry and Laboratory Medicine data for PTH (within the subject biological variation [CVi] = 15.7% and between-subject biological variation [CVg] = 23.5%) sets the desirable specification for bias at less than 7.1%. Cavalier (24) found that the median desirable bias was 8.8% in healthy subjects and 15.9% in haemodialysed patients. In this study, the estimated bias for CKD stages 3b to 5D that we obtained exceeded the desirable performance specification, specifically at its upper reference limit which is the important level in determining high-turnover CKD-MBD (Table 4). Thus, iPTH and bio-PTH cannot be used interchangeably, as although they had good agreement, they also had a huge bias.

KDIGO recommended that CKD-MBD diagnosis be established based on a biochemical trend rather than a single measurement and for the clinician to be informed of changes in the method used. Shifting from iPTH to bio-PTH could cause misinterpretation due to lower bio-PTH levels and the different target range used to determine CKD-MBD. Current guidelines on managing CKD-MBD do not yet include the target value when using bio-PTH assay. The PTH target range of the Kidney Disease Outcomes Quality Initiative guideline of 150 pg/mL–300 pg/mL was based on studies that used the Allegro iPTH assay, which is no longer available for standardisation. Few studies have established target values for bio-PTH assay. Cavalier et al. (24) set the reference interval at 8 pg/mL–45 pg/mL for normal healthy individuals, whereas for haemodialysed patients, Beko et al. (25) set the target range at 85 pg/mL–258 pg/mL and the Japanese Society of Dialysis Therapy (JDST) Group recommends maintaining the PTH concentration between 35 pg/mL and 150 pg/mL.

If the third-generation bio-PTH assay will be implemented, classification of a patient using current established target ranges might not be optimal and could be misleading. Misclassification of patients was revealed revealed when Elecsys iPTH was switched to Elecsys bio-PTH (1–84), where 10% of the patients who were classified as above the therapeutic range came within the recommended therapeutic range, whereas another 14% who initially belonged to the group within the recommended therapeutic range came within the subtherapeutic range (25). Taniguchi et al. (26) showed that when the PTH concentration of the total subject population was measured using bio-PTH assay, 18% of it was misclassified into different JDST classifications.

Two iPTH assays from different manufacturers were shown to yield different results on haemodialysed patients (27). As iPTH assays are not standardised, a change of methods when a haemodialysed patient is transferred to another dialysis centre may change the PTH concentration result, which may alter the therapeutic decision. Bio-PTH assay has the possibility of standardisation. When assays were standardised based on the World Health Organization (WHO) International PTH standard 95/646, the inter-method variability was significantly reduced and the result did not change the classification of the patient following the target range in the guideline (28). In our study, hypocalcaemia and vitamin D deficiency appeared as early as in CKD stage 4, and despite the phosphate, the PTH and ALP levels still fell within the normal reference intervals. A study reported that serum 25(OH)D started to decline as early as in CKD stage 2 and that the deficiency began before hyperphosphatemia became prominent in CKD-MBD (25). We found vitamin D deficiency among CKD stages 3, 4 and 5D patients, and Kota et al. (29) observed the association of < 30 ng/mL of serum vitamin D and an increasing PTH level with lower bone mineral density. These findings are consistent with those of Coen et al. (30) of the association of a low bone turnover with a < 20 ng/mL 25(OH)D level. However, it was found (31) that peripheral confounding factors need to be considered in determining vitamin D deficiency, including inadequate sunlight exposure and effects of pregnancy, skin color, aging, and drugs such as antiepileptics and cholestyramine.

KDOQI and KDIGO recommend considering active vitamin D analogue when the 25(OH)D level is < 30 ng/mL in CKD stages 3 and 4 accompanied by secondary hyperparathyroidism features. Consistent with this guideline, current clinical practice prescribes vitamin D analogue, calcimimetics and phosphate binders when the patient had entered the dialysis stage or when CKD-MBD is clinically and biochemically suggested. However, our data on dialysed patients showed that they had elevated PTH, phosphate, CTX, P1NP with hypocalcemia and vitamin D deficiency, which may require treatment optimisation.

Our correlation study showed that PTH assays had a significant correlation with bone turnover markers, which was also observed in other studies (9, 12). These findings validate the association of PTH assays with bone turnover markers in CKD-MBD. However, we found that both PTH assays had a high positive correlation only with CTX and P1NP, whereas they had only a moderate positive correlation with phosphate and a low to negligible positive correlation with calcium, ALP and 25(OH)D (21).

In Hu et al. (32), the CTX reference interval was set at 0.112 ng/mL–0.497 ng/mL, and the P1NP reference interval, at 13.72 ng/mL–58.67 ng/mL. Based on these data, we found that CTX and total P1NP increase beginning in CDK stage 4 and are significantly elevated when the patient requires dialysis.

Although both PTH assays had a high correlation with CTX and P1NP in this study, we observed that iPTH assay had a slightly higher *r*-value than bio-PTH assay. However, bone biopsy is needed to validate the discriminative ability of both PTH assays to determine bone turnover. Salam et al. (33) found that when iPTH is combined with other bone turnover markers, it has better discriminative ability to determine high bone turnover than a similar combination with bio-PTH. However, their study also concluded that no single or combined biomarker was robust enough to diagnose low, normal and high bone turnovers.

This study had a few limitations. The sample size was small, and the patients’ samples were taken once and not serially. Bone mass assessment and bone biopsies were also lacking. Moreover, confounding factors of the vitamin D level were not considered, which might have contributed to variations in the vitamin D measurement.

## Conclusion

Our study found that bio-PTH assay agrees well with iPTH assay, but they cannot be used interchangeably due to the unacceptable large bias in later-stage CKD. In our opinion, bio-PTH assay has the advantage of potential standardisation. Further evaluation of the correlation of bio-PTH assay with CKD-MBD diagnosis and gold-standard bone histomorphometry is needed to determine if bio-PTH assay is a better CKD-MBD marker. To use bio-PTH, a target value for it must be urgently established, particularly among CKD-MBD patients. Different cutoff points should be used to categorise CKD-MBD patients based on their PTH measurement for their better management. Both PTH assays were also correlated with bone turnover markers, especially with CTX and P1NP, but further assessment correlating them with bone biopsies is needed to determine their ability to discriminate CKD-MBD.

## Figures and Tables

**Table 1:** Baseline characteristics of study subjects

Parameter	Total mean (SD)/median [IQR]	CKD stage 3 mean (SD)/median [IQR]	CKD stage 4 mean (SD)/median [IQR]	CKD stage 5D mean (SD)/median [IQR]
N	180	60	60	60
eGFR (CKD-EPI)	21 [29]	42 [14]	21 [7]	6 [4]
Age (years old)	58 [18]	60 [16]	61 [13]	52 [27]
Intact PTH (pg/L)	113 [223]	58 [62]	94 [85]	378 [481]
Biointact PTH (pg/L)	103 [143]	55 [67]	85 [76]	252 [280]
Serum calcium (mmol/L)	2.19 (0.23)	2.24 (0.20)	2.18 (0.15)	2.15 (0.30)
Serum phosphate (mmol/L)	1.43 (0.46)	1.22 (0.25)	1.38 (0.33)	1.69 (0.61)
Serum alkaline phosphatase (U/L)	129 (122)	94.75 (29.32)	104.86 (38.27)	118.5 [68.0]
Serum P1NP (ng/mL)	139 [275]	75 [77]	125 [92]	595 [904]
Serum CTX (ng/mL)	1.25 [1.34]	0.71 [0.55]	1.14 [0.64]	2.60 [5.42]
Serum 25(OH)D (ng/mL)	20 [20]	22 [20]	16 [16]	19 [15]

Notes: CTX = C-terminal telopeptide collagen; P1NP = amino-terminal of type 1 pro collagen; 25(OH)D = 25-hydroxyvitamin D

**Table 2:** Agreement between iPTH and bio-PTH level according to CKD stages using ICC

	ICC^a^	95% Confidence interval		*P*-value

		Lower bound	Upper bound	
All CKD stages	0.83^b^	0.79	0.86	< 0.001
CKD stage 3	0.93^b^	0.89	0.95	< 0.001
CKD stage 4	0.91^b^	0.87	0.94	< 0.001
CKD stage 5D	0.75^b^	0.168	0.902	< 0.001

Notes: Two-way random effects, absolute agreement, single rater/measurement;

a type A intraclass correlation coefficients using an absolute agreement definition, value taken of single measures;

b the estimator is the same, whether the interaction effect is present or not

**Table 3:** Average concentrations (pg/L) using Passing-Bablok regression analysis and Bland-Altman analysis of the iPTH assay compared to bio-PTH assay for CKD stages 3–5D

CKD stage	Passing-Bablok regression		Bland-Altman analysis	
	
	Intercept (95% CI)	Slope (95% CI)	Mean difference	LOA
All three stages	15.80 (14.23, 19.05)	0.64 (0.61, 0.66)	−71.486	−346.91 to 203.94
3	8.04 (5.29, 12.36)	0.87 (0.76, 0.94)	−3.24	−47.40 to 40.93
4	13.66 (6.2, 17.05)	0.80 (0.75, 0.85)	−20.69	−108.36 to 66.97
5D	24.12 (17.23, 31.38)	0.58 (0.55, 0.61)	−190.53	−560.50 to 179.44

Notes: CI = confidence interval; CKD = chronic kidney disease

**Table 4:** Bias estimation in comparison to the desirable allowable bias specification

CKD stage	Regression formulae	KDIGO target value iPTH (24)		Bio-PTH target value (25)		$\text{Percentage bias }(\%)\frac{\left(\text{Y value}-\text{target value}\right)}{\text{Target value}}\times 100$				Desirable bias (EFLM) (%)	Desirable bias (24) (%)
		
		LL	UL	LL	UL	iPTH		Biointact PTH			
	
						LL	UL	LL	UL		
3	Y= 0.87x + 8.04	100	451	85	258	5.0	11.2	3.5	9.8	7.1	8.8
4	Y = 0.80x + 13.7	100	451	85	258	12.0	17.0	3.9	14.7	7.1	8.8
5D	Y = 0.58x + 24.1	100	451	85	258	17.9	36.7	13.6	22.7	7.1	15.9

Notes: KDIGO = Kidney Disease Improving Global Outcomes; iPTH = intact PTH; LL = lower target limit; UL = upper target limit

**Table 5:** Correlation between iPTH and bio-PTH levels with serum bone parameters using Spearman rho correlation analysis

Laboratory values	iPTH assay		Bio-PTH assay	
	
	r	*P*-value	r	*P*-value
Serum CTX	0.755	< 0.001	0.743	< 0.001
Serum P1NP	0.726	< 0.001	0.709	< 0.001
Serum 25(OH)D	−0.199	0.007	−0.187	0.012
Serum calcium	−0.350	< 0.001	−0.337	< 0.001
Serum phosphate	0.522	< 0.001	0.505	< 0.001
Serum alkaline phosphatase	0.458	< 0.001	0.466	< 0.001

Notes: CTX = C-terminal telopeptide collagen; P1NP = amino-terminal of type 1 procollagen; 25(OH)D = 25-hydroxyvitamin D
